# Improved biosensing of *Legionella* by integrating filtration and immunomagnetic separation of the bacteria retained in filters

**DOI:** 10.1007/s00604-023-06122-1

**Published:** 2024-01-09

**Authors:** Melania Mesas Gómez, Bárbara Molina-Moya, Bárbara de Araujo Souza, Maria Valnice Boldrin Zanoni, Esther Julián, José Domínguez, Maria Isabel Pividori

**Affiliations:** 1 Grup de Sensors i Biosensors, Departament de Química, Universitat Autònoma de Barcelona, Bellaterra, Spain; 2 Biosensing and Bioanalysis Group, Institute of Biotechnology and Biomedicine, Universitat Autònoma de Barcelona, 08193 Bellaterra, Spain; 3 Institut d’Investigació Germans Trias i Pujol (IGTP), 08916 Badalona, Spain; 4 CIBER Enfermedades Respiratorias, Instituto de Salud Carlos III, Departament de Genètica i Microbiologia, Universitat Autònoma de Barcelona, Bellaterra, Spain; 5 Department of Analytical Chemistry, Institute of Chemistry, UNESP, Universidad Estadual Paulista, Araraquara, SP Brazil; 6 Departament de Genètica i Microbiologia, Universitat Autònoma de Barcelona, Bellaterra, Spain

**Keywords:** Electrochemical immunosensor, Square wave voltammetry, Water-borne bacteria, Solid-phase separation, Magnetic particles, Preconcentration strategy

## Abstract

A novel approach is presented that combines filtration and the direct immunomagnetic separation of the retained bacteria *Legionella* in filters, for further electrochemical immunosensing. This strategy allows for the separation and preconcentration of the water-borne pathogen from high-volume samples, up to 1000 mL. The limit of detection of the electrochemical immunosensor resulted in 100 CFU mL^−1^ and improved up to 0.1 CFU mL^−1^ when the preconcentration strategy was applied in 1 L of sample (10^3^-fold improvement). Remarkably, the immunosensor achieves the limit of detection in less than 2.5 h and simplified the analytical procedure. This represents the lowest concentration reported to date for electrochemical immunosensing of *Legionella* cells without the need for pre-enrichment or DNA amplification. Furthermore, the study successfully demonstrates the extraction of bacteria retained on different filtering materials using immunomagnetic separation, highlighting the high efficiency of the magnetic particles to pull out the bacteria directly from solid materials. This promising feature expands the applicability of the method beyond water systems for detecting bacteria retained in air filters of air conditioning units by directly performing the immunomagnetic separation in the filters.

**Graphical abstract:**

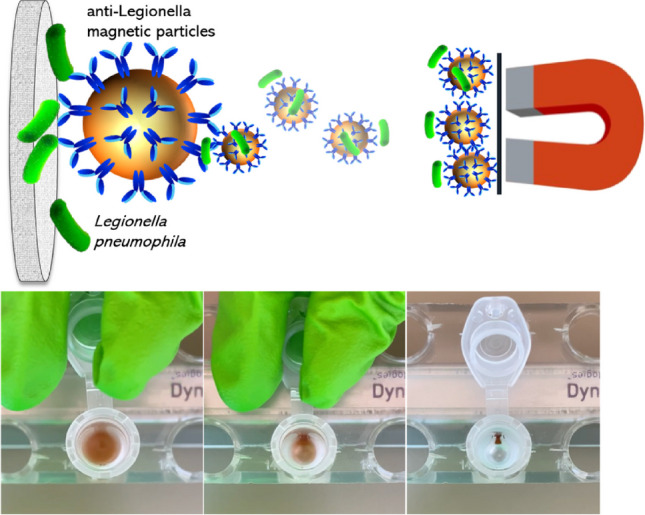

**Supplementary Information:**

The online version contains supplementary material available at 10.1007/s00604-023-06122-1.

## Introduction

*Legionella* is the primary cause of a severe form of pneumonia [1], being *Legionella pneumophila* serogroup 1 (Lp1) the major responsible of infections in humans [2]. *Legionella* can grow in natural and artificial water systems with little or no circulation [3, 4]. Transmission typically occurs by inhalation of contaminated aerosols or mist, with no person-to-person spread, being the infective dose as low as a single colony. Outbreaks are often associated with poorly maintained artificial water systems, such as cooling towers, evaporative condensers linked to air conditioning and industrial cooling, as well as hot and cold-water systems [5], among many others sources [6–9]. Hospitals can also be sources of infection [10], as well as contaminated drinking water. The prevention of legionellosis relies on implementing control measures to minimize the growth of *Legionella* and the dissemination of aerosols. Besides the good maintenance of devices, regular cleaning, and disinfection [5], prevention strategies involve periodic on-site testing of potential outbreaks. The specific approaches for *Legionella* infection prevention and monitoring may vary depending on local regulations and geographic locations [11, 12]. Currently, the most widely used methods for detection involve time-consuming culture-based techniques followed by molecular methods. The limit of detection (LOD) for *Legionella* in different sources also vary depending on the regulations stablished by each country. For instance, the European Technical Guidelines for the Prevention, Control, and Investigation of Infections caused by *Legionella* species recommend testing samples in accredited laboratories with a LOD of 100 CFU L^−1^ [13]. However, there is a general agreement that corrective actions should be taken when concentrations reach 10,000 CFU L^−1^ [11]. For instance, cooling towers and spa pools require minor interventions or disinfection, typically at LODs higher than 100 CFU L^−1^. Moreover, in cooling towers actions need to be taken from 10^5^ to 10^6^ CFU L^−1^ according to the American Industrial Hygiene Association (AIHA) Guidance on *Legionella* in building water systems [14].

The gold-standard method for the detection of *Legionella* in water samples includes high-volume sample collection of up to 1 L, followed by membrane filtration as a preconcentration step, isolation on culture plates for 10 days, and confirmation using sub-culturing according to ISO 11731. Alternatively, some commercial kits, as Legiolert® test (IDEXX Laboratories, Westbrook, ME, USA) based on enzymatic detection technology, use lower volumes of 1 or 100 mL providing results in 7 days [15]. Lateral Flow Tests, such as Duopath® (Merck) or Hydrosense® (Albagaia, Edinburgh, UK), offer rapid visual redouts, but either require several days of enrichment to reach the LOD required by the legislation or yield semiquantitative results. To improve LODs without time-consuming pre-enrichment steps, the only alternative involves DNA amplification rather than bacterial growth. Commercial options for this approach include the microproof® *Legionella* Quantification LyoKit from BIOTECON Diagnostics GmbH (Potsdam, Germany) or the iQ-Check *Legionella* Real-Time PCR Kits from BIO-RAD (Bio-Rad Laboratories Inc, California, USA).

In order to reduce the time of analysis, it becomes imperative that emerging *Legionella* rapid testing technologies eliminate the necessity for pre-enrichment or DNA amplification procedures. As an alternative of amplification procedures, a highly efficient preconcentration strategy that combines filtration with immunomagnetic separation of the bacteria retained in the filters was successfully integrated to an immunosensing device. This novel approach has resulted in a significant improvement in the limit of detection compared to previously reported immunosensing devices [16–21]. This approach enables the separation and preconcentration of pathogens from large sample volumes, typically 100–1000 mL, where immunomagnetic separation alone would not be feasible. The electrochemical immunosensor developed in this work is optimized and compared with the culturing methods in terms of the analytical features. Additionally, the performance of direct immunomagnetic separation of bacteria retained on various filtering materials is also assessed, highlighting the high efficiency of the magnetic particles to pull out the bacteria from solid materials.

## Experimental section

### Instrumentation

A complete filtration system of 25 mm (Product no. 073-0Q7724, Scharlab) was used for filtration. The filters assessed were polycarbonate cyclopore track etched (Catalogue no. 7060-2504, Whatman), nylon (Catalogue no. 7404-002, Whatman), cellulose acetate (Catalogue no. 10404006, Whatman), cellulose nitrate (Catalogue no. 10401106, Whatman), and mixed cellulose ester (Ref. HAWP02500, Merck Millipore Ltd), in all instances 0.45-μm pore size and 25-mm diameter. The magnetic actuation was achieved with the 16-tube magnet (Product no. 12321D, Thermo Fisher Scientific). The electrochemical readout was achieved on carbon screen-printed electrodes (ref. DRP-C110) using a portable bipotentiostat DRP-STAT200 operated by DropView 200 for instrument control and data acquisition (Dropsens, Spain). The scanning electron microscope (SEM) images were taken with the EVO MA-10 (with EDS Detector, Oxford LINCA).

### Chemicals and biochemicals

Different set of buffers were used for specific procedures in the experiments, and their composition is described in S1 (Supp. data). All buffers were prepared from chemicals of analytical grade purchased from Merck and Sigma and using milliQ water. The reagents used for the electrochemical measurement includes hydroquinone (Ref. H9003) and hydrogen peroxide 30% solution (Ref. 31642, Sigma-Aldrich).

The anti-*L. pneumophila* monoclonal antibody (G90A) from mouse (Catalogue no. MA5-18213, Invitrogen) was immobilized on tosyl-activated magnetic particles (MPs, Dynabeads M-450 Tosylactivated, Product no. 14203, Invitrogen). The tailored modification of the antibody on the MPs is described in detail in S2 (Supp. data). The anti-*Legionella* polyclonal antibody labelled with horseradish peroxidase (HRP) enzyme from rabbit was used as a secondary antibody (Catalogue no. PA1-73141, Invitrogen). The strains used were *Legionella pneumophila* (serogroup 1. Philadelphia 1, ATCC 33152), and for the specificity study, *Pseudomonas aeruginosa* (ATCC 15442), *Klebsiella pneumoniae* (Schroeter) Trevisan (ATCC BAA-1705), *Mycobacterium fortuitum* (ATCC 6841), *Enterobacter* spp. (used as control by Sociedad Española de Enfermedades Infecciosas y Microbiología Clínica), *Escherichia coli* (ATCC 10536), and *Salmonella choleraesuis* (ATCC 13311).

### *Legionella pneumophila* culture

*L. pneumophila* strain was grown in selective solid culture plates (*Legionella* MWY Selective Agar, Product no. 10482513, Thermo Scientific) at 37 °C for 72 h. The bacteria were inoculated to distilled water and the solution was adjusted to an OD 0.370 at 600 nm, approximately equivalent to 10^7^ CFU mL^−1^. The concentration of *Legionella* samples was calculated for each experiment by colony counts verification in solid culture, as shown in Figure S1 (Supp data).

### Evaluation of the immunomagnetic separation by scanning electron microscopy

To assess the performance anti-Legionella-MPs for binding *Legionella*, scanning electron microscopy was performed to visualize the interaction between the modified MPs and the bacteria. A volume of 100 μL of anti-*Legionella*-MPs at 10^7^ MP mL^−1^ was incubated with 1 mL of *L. pneumophila* at 10^7^ CFU mL^−1^ approximately, for 1 h at room temperature (RT) with agitation at 750 rpm. Afterwards, the supernatant was discarded, and the sample was washed three times with 1 mL of washing buffer. The sample was resuspended in 4 mL of PBS and it was filtered in a polycarbonate membrane to retain the modified MPs capturing the bacteria. Then, the filter was treated as previously described [16].

### Electrochemical magneto immunosensing. Specificity study

The electrochemical magneto immunosensing procedure has been previously developed by our research group in other applications [16–19]. Briefly, involves (i) the immunomagnetic separation (IMS), (ii) the incubation with the label antibody, and (iii) the electrochemical readout. The optimization of the reagents including anti-*Legionella*-MPs (ranging from 10^4^ to 10^7^) and anti-*Legionella*-HRP antibody (from 1/250 to 1/2000) are described in S3 (Supp. data). Briefly, 100 μL of sample containing *Legionella* was incubated with 100 μL of MPs at 10^7^ MP mL^−1^ in a final volume of 1 mL with PBS for 1h under gentle rotation. After a washing step, the sample was incubated with 200 μL of anti-*Legionella*-HRP at a dilution of 1/500 for 30 min at 900 rpm and RT. After washing, the electrochemical readout was performed by resuspending the MPs in 40 μL of ePBS and 18 μL of electrochemical readout substrate solution. After 2 min of enzymatic reaction, the solution was dropped to the screen-printed electrode and the electrochemical readout was done based on the square wave voltammetry technique (SWV). More details about the SWV measurements are provided in S5 (Supp. data). The LOD of the electrochemical magneto immunosensing procedure was calculated by processing a calibration plot ranging from 0 to 3.6 × 10^5^ CFU mL^−1^.

The specificity study was performed by challenging the immunosensor with other water-borne bacteria at high concentration (10^7^/10^8^ CFU mL^−1^) such as *Pseudomonas aeruginosa*, *Klebsiella pneumoniae*, *Mycobacterium fortuitum*, *Enterobacter* spp., *Escherichia coli*, and *Salmonella choleraesuis* in a final volume of 1 mL and proceeded as above.

### Novel preconcentration method: study of the filtering material

**Fig. 1:**
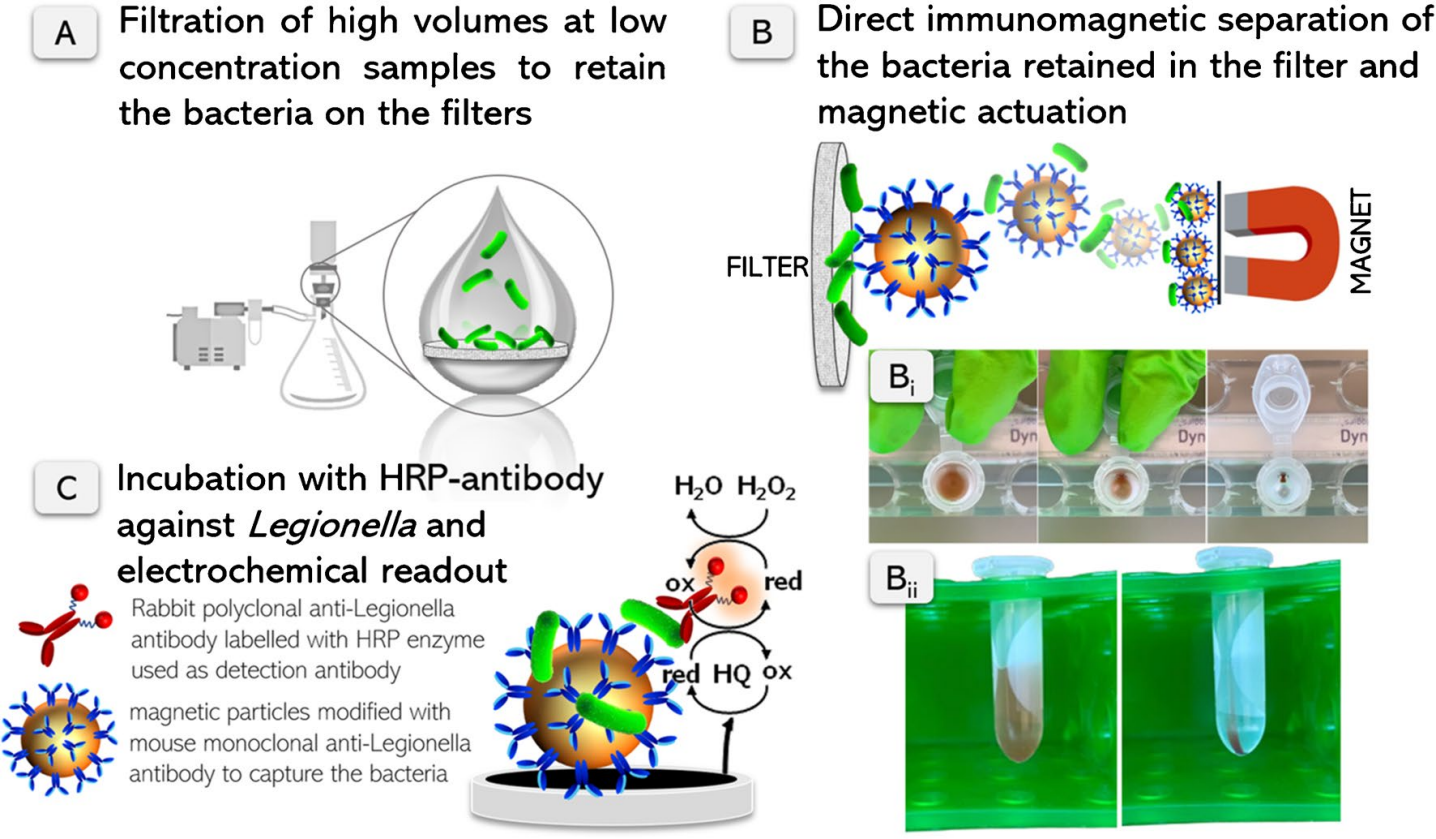
Schematic representation of **A** filtration, **B** immunomagnetic separation of the bacteria retained in the filters and magnetic actuation, followed by **C** electrochemical immunosensing for the detection of *Legionella pneumophila* in water samples. In **B**, experimental details are presented, showing how the filter is positioned on the Eppendorf tube for immunomagnetic separation, along with the evaluation of magnetic actuation efficiency on the filters. The photos in (Bi) were captured in 1-s frame sequences. More details are presented in S6 (Supp. data)

The procedure combines three steps as described in Fig. 1: (i) filtration of large volumes of sample (typically 100 or 1000 mL), followed by (ii) immunomagnetic separation of the bacteria retained in the filter and magnetic actuation, (iii) electrochemical immunosensing, as described above.

The experimental details are shown in Figure S6 (Supp. data). After filtration from 100 to 1000 mL sample under vacuum, the 25-mm diameter filter was placed on a 2.0-mL tube. 100 μL of anti-*Legionella*-MPs at 10^7^ MP mL^−1^ and 900 μL of PBS were then added to the filter and incubated under gentle rotation at RT for 1 h. Then, the MPs with the captured *Legionella* were recovered under magnetic actuation and washed for 3 min at 900 rpm and RT. After that, 200 μL of the anti-*Legionella*-HRP antibody was added to the modified MPs and incubated for 30 min at 900 rpm and RT. After washing, the electrochemical readout was performed as described above.

In order to validate the approach, different materials were assessed. Polycarbonate (PC), nylon (NY), cellulose acetate (CA), cellulose nitrate (CN), and mixed cellulose ester membranes (MCE) were selected because of their widely use in microbiological filtration techniques with a 0.45-μm porous size and 25-mm diameter. For this study, 100 mL *Legionella* samples at the same concentration (2 × 10^3^ CFU mL^−1^) as well as the negative control were filtered using each membrane and submitted to downstream analysis, as above.

### Statistical analysis

Statistical analyses were conducted using Prism v 10.0.1 (GraphPad, San Diego, USA). Calibration curves were fitted using nonlinear regression with a four-parameter logistic curve 4PL, defined by the equation:

$$Y=D+\left(A-D\right)/\left[1+\left(C/ IC50\right)\hat{\phantom{0}} HillSlope\right]$$

where: Y: represents the electrochemical signal; D: stands for the lower asymptote, representing the response at very low concentrations; A: represents the upper asymptote, signifying the response at high concentrations; IC50: denotes the inflection point or concentration at the midpoint of the curve; HillSlope: signifies the slope parameter.

The limit of detection (LOD) was determined by analyzing negative control samples to calculate the mean value and standard deviation (SD). Subsequently, a one-tailed t-test was applied at a 95% confidence level to establish the cut-off value. The LOD was then derived by interpolation of this cut-off value using the 4PL equation, followed by the calculation of the corresponding *Legionella* concentration.

### Biosafety considerations

All experiments were performed in a Biosafety Class 2 environment required for the handling of *L. pneumophila*. All biological waste generated from the experiments were disposed in accordance with the local regulations for handling biohazards.

## Results and discussion

### Evaluation of the immunomagnetic separation by scanning electron microscopy

**Fig. 2:**
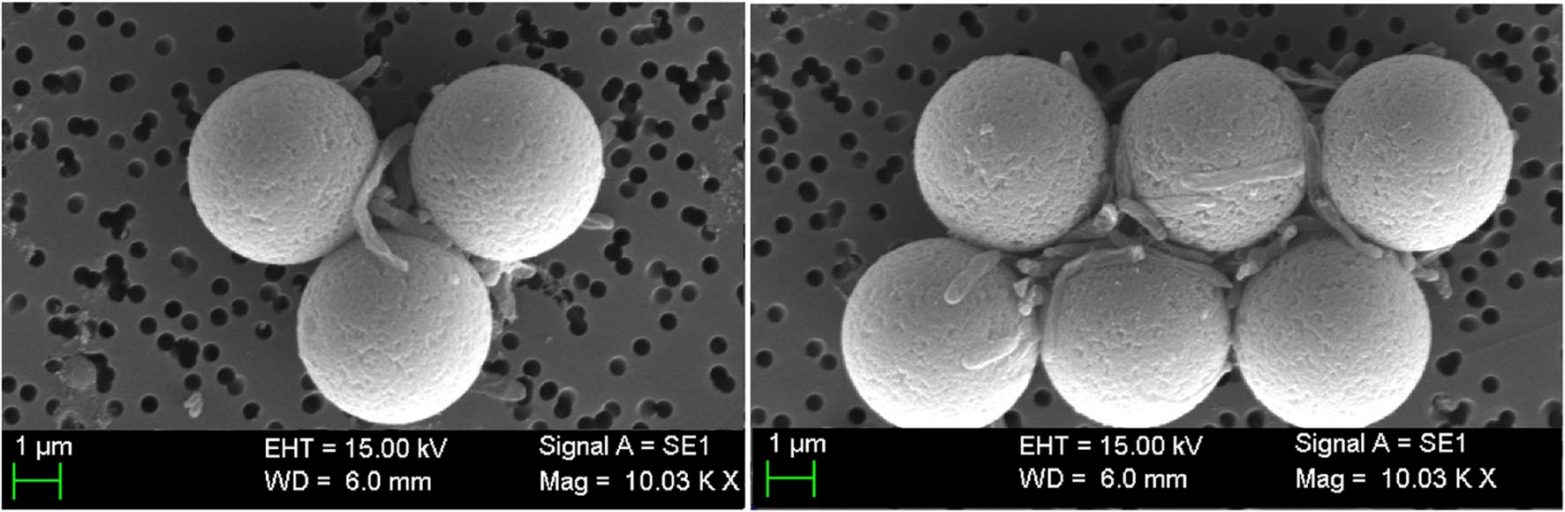
Evaluation of the IMS by SEM at a *L. pneumophila* concentration of 10^7^ CFU mL^−1^. The images show the *L. pneumophila* cells attached to the magnetic beads. In all cases, identical acceleration voltage (15 KV) was used

Following the incubation of the *Legionella*-containing sample with anti-*Legionella* magnetic particles (anti-*Legionella*-MPs), the sample was observed by scanning electron microscopy (SEM) to confirm the effectiveness of the immunomagnetic separation. In Fig. 2, it is confirmed that tosyl-activated magnetic particles were coated with anti-*Legionella* monoclonal antibodies to enhance the interaction between the particles and the bacteria. The images clearly demonstrate the successful binding of the modified magnetic particles to the *L. pneumophila* bacteria, resulting in the formation of clusters due to the polyvalency of both the bacteria and the magnetic particles.

### Electrochemical magneto immunosensing: specificity study

The conditions for the electrochemical immunosensing were previously optimized as described in S3 (Supp. data). From the results, a concentration of 10^7^ anti-*Legionella*-MPs mL^−1^ and anti-*Legionella*-HRP antibody 1/500 was used in all further experiments. Figure S4 (Supp. data), shows the calibration plot from 0 to 3.6 × 10^5^ CFU mL^−1^ for the determination of the *L. pneumophila* with the electrochemical immunosensor without the integration of the novel preconcentration method. The data was fitted with a non-linear regression (Sigmoidal 4PL, GraphPad Prism Software v 10.0.1, *R*^2^= 0.9837) and the LOD was calculated, resulting in a value of 100 CFU mL^−1^. As expected, this LOD is comparable with a magneto-actuated immunosensor previously reported by our research group but for other bacteria [17, 22].

**Fig. 3:**
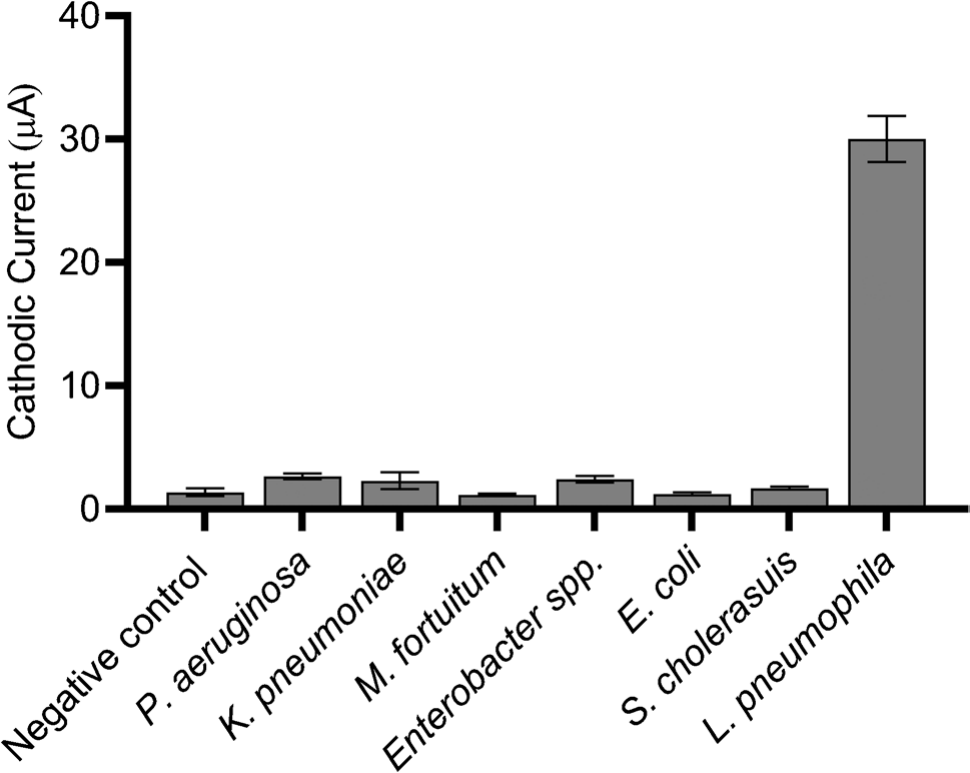
Specificity study for *L. pneumophila*, including a negative control, and interferents at high concentration, including *Pseudomonas aeruginosa* (2 × 10^8^ CFU mL^−1^), *Klebsiella pneumoniae* (2 × 10^8^ CFU mL^−1^), *Mycobacterium fortuitum* (2 × 10^5^ CFU mL^−1^), *Enterobacter* spp. (2 × 10^8^ CFU mL^−1^), *Escherichia coli* (2 × 10^8^ CFU mL^−1^), and *Salmonella choleraesuis* (3 × 10^8^ CFU mL^−1^). *L. pneumophila positive control* (8 × 10^4^ CFU mL^−1^) is also included. The concentrations of the viable bacteria were determined by culturing in solid media and CFU counting. The error bars show the standard deviation for *n*=3

The specificity of the electrochemical biosensor towards a high concentration of other water-borne bacteria was also studied, and the results are shown in Fig. 3. The signal of the negative control containing 0 CFU mL^−1^ of *Legionella* (negative control) was found to be comparable with all the interferents bacteria samples (*p* < 0.05), including *Pseudomonas aeruginosa*, *Klebsiella pneumoniae*, *Mycobacterium fortuitum*, *Enterobacter* spp., *Escherichia coli*, and *Salmonella choleraesuis*, suggesting a good specificity for the assay.

The electrochemical magneto immunosensor was specifically designed for the determination of *Legionella* by utilizing a monoclonal antibody that targets the lipopolysaccharides of *L. pneumophila* strains. As a result, other bacteria are not detected, ensuring specificity for *Legionella* determination. It is important to note that the immunosensor primarily targets *Legionella pneumophila* serogroup 1, which is known for its high virulence and prevalence in cooling towers and distribution systems, making it the key strain responsible for most infections.

### Novel preconcentration method: study of the filtering material

**Fig. 4:**
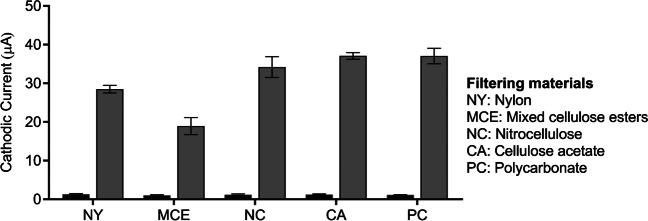
Study of the filtering material by the filtration of a 100 mL sample containing *Legionella* (2 × 10^3^ CFU mL^−1^) as well as the negative controls. All the filters were 0.45 μm pore size and 25 mm of diameter. The filtering materials tested were nylon (NY), mixed cellulose ester (MCE), cellulose nitrate or nitrocellulose (NC), cellulose acetate (CA), and polycarbonate (PC). The concentrations of the viable bacteria were determined by culturing in solid media and CFU counting. The error bars show the standard deviation for *n*=3

The LOD achieved by the electrochemical magneto immunosensor (1 × 10^2^ CFU mL^−1^) are above the limits required by most legislations. Accordingly, and to overcome time-consuming classical pre-enrichment [17] or DNA amplification [16], a novel preconcentration method is proposed, combining filtration and direct immunomagnetic separation (IMS) of the bacteria retained in the filters, as depicted in Fig. 1, to match the limits required by the legislation. Selecting the appropriate filter material is crucial to ensure a swift filtration workflow and minimize nonspecific adsorption of bacteria on the filters. This is essential for subsequent immunomagnetic separation and effective magnetic actuation of the retained bacteria in the filters. Accordingly, a first study was performed with a set of filters of different materials. There is a wide range of commercially available membranes with low retention rates and different properties. Filtering materials commonly used in microbiology were selected in this study, including polycarbonate, nylon, cellulose acetate, cellulose nitrate and mixed cellulose esters, and the results are shown in Fig. 4. They share a hydrophilic surface to avoid an excessive retention of the bacteria, and thus promoting the capture by the anti-*Legionella*-MPs directly in the filter. Moreover, the hydrophobic nature of the outer surface of *Legionella* [23, 24] makes easier pull-off the trapped bacteria in hydrophilic surfaces.

According to the results shown in Fig. 4, no significant differences in the current intensities by using polycarbonate (PC), cellulose nitrate, and cellulose acetate (CA) filters were obtained (*p* > 0.05). The use of PC membranes in the filtration processes is recommended by some regulations, for instance the standard ISO11731:2017, and several studies support their efficacy as they provide the best recovery rates of this bacterium [25]. Some of the advantages of PC track-etched membranes are their precision in the cylindrical pores providing a very accurate size cut-off, and their low nonspecific protein binding, similar to the CA membranes, which also provided good results. In contrast, nylon and mixed cellulose ester materials gave lower intensity currents in comparison with PC as a reference material. The high protein binding capacity of these filters could cause a highly retention of the bacteria in the filter, thus hindering the immunomagnetic capturing and resulting in lower preconcentration yields. Apart from the material, porosity, pore size, and the protein binding capacity, among other features, other variables such as electrostatic charges can also influence in the filtration and the magnetic actuation. Although PC, CA, and cellulose nitrate membranes can all be considered as good candidates for the preconcentration strategy, the workflow time was different for each filtering material (Table S6, Supp. Data), providing the polycarbonate improved features, such as faster filtration rates, resulting in less than 1 min for the filtration of 100 mL sample and less than 10 min for the filtration of 1 L sample. In the case of the cellulose nitrate and cellulose acetate filters, the workflow time was higher. According to these results, PC filters were selected in all further experiments.

**Fig. 5:**
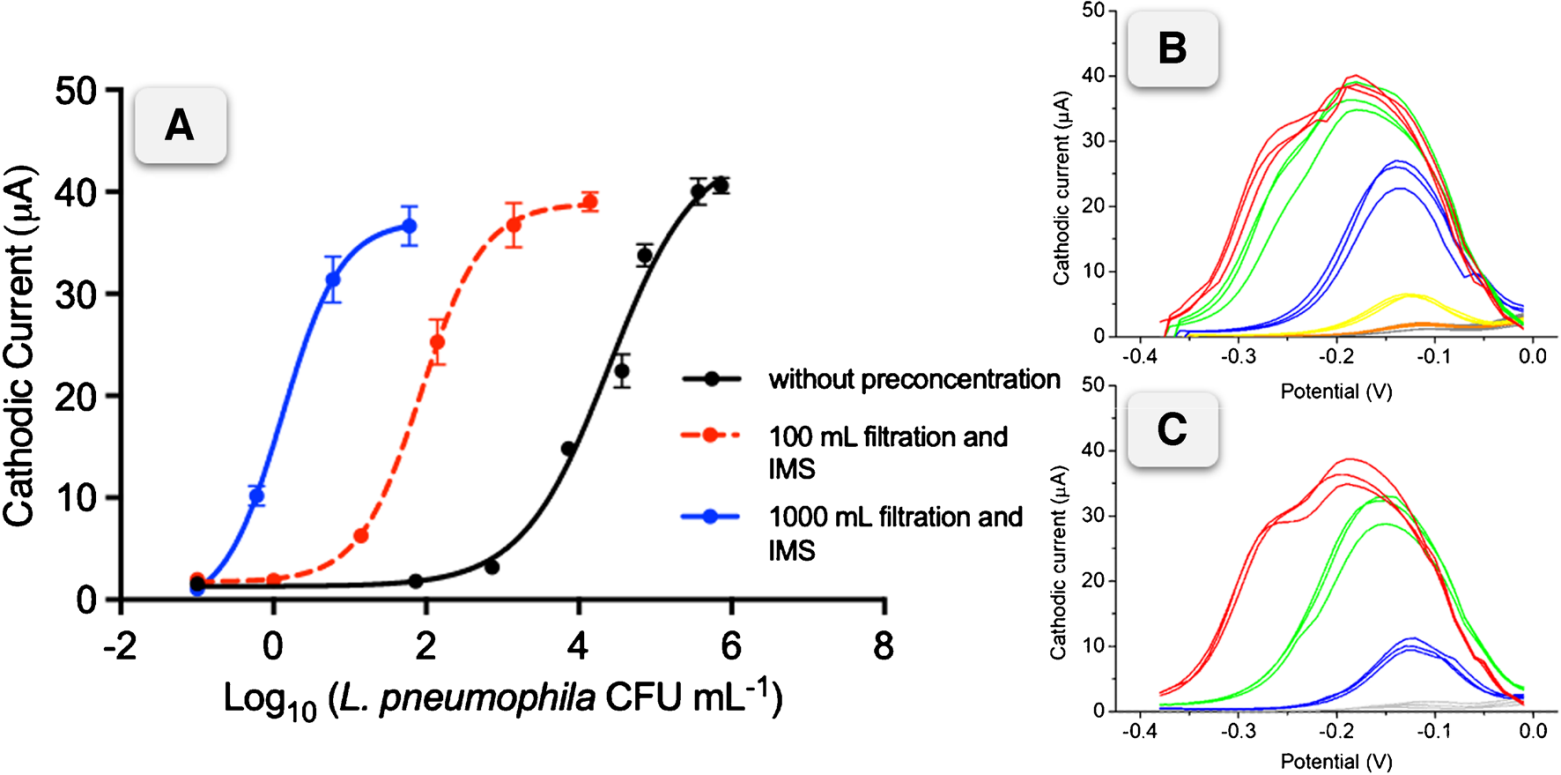
**A** Calibration plot for the magneto-actuated electrochemical immunosensor in water samples. The black solid line shows the calibration plot ranging from 0 to 7 × 10^5^ CFU mL^−1^ without the integration of the novel preconcentration method (*R*^2^= 0.9886). The red dotted line shows the calibration plot ranging from 0 to 1 × 10^4^ CFU mL^−1^ (*R*^2^= 0.9950) with preconcentration by filtering 100 mL samples. Raw data obtained from the SWV measurements is shown in **B**. The blue solid line shows the calibration plot ranging from 0 to 60 CFU mL^−1^ (*R*^2^= 0.9923) by filtering 1000 mL samples. Raw data obtained from the SWV measurements is shown in **C**. After filtration, IMS and electrochemical immunosensing are performed in both cases. The concentrations of the viable bacteria were determined by culturing in solid media and CFU counting. The error bars show the standard deviation for *n*=3

The calibration plot integrating the novel preconcentration method (by filtering 100 mL of sample and IMS), and further electrochemical immunosensor is shown in Fig. 5 as red dotted line, for the determination of the *L. pneumophila* from 0 to 1 × 10^4^ CFU mL^−1^. The data was fitted with a non-linear regression (Sigmoidal 4PL, GraphPad Prism Software v 10.0.1, *R*^2^= 0.9950) and the LOD was calculated, resulting in a value of as low as 2 CFU mL^−1^. Compared with the electrochemical biosensor without the integration of the preconcentration method (Fig. 5, black solid line), an improving of two orders of magnitude was achieved, from 1 × 10^2^ CFU mL^−1^ to 2 CFU mL^−1^. According to some legislations, the approach is useful to detect the threshold of 10,000 CFU L^−1^ established for corrective actions, especially in cooling towers [14, 26]. However, certain regulations may require even lower LODs. In order to achieve a further improvement in the LOD, integrating the novel preconcentration method (by filtering 1000 mL of sample and IMS), and further electrochemical immunosensor is shown in Fig. 5 as blue solid line, for the determination of the *L. pneumophila* from 0 to 60 CFU mL^−1^. The data was fitted with a non-linear regression (Sigmoidal 4PL, GraphPad Prism Software v 10.0.1, *R*^2^= 0.9923) and the LOD was calculated, resulting in a value of as low as 0.1 CFU mL^−1^ (100 CFU L^−1^). Interestingly, the filtration and immunomagnetic separation of 1000 mL of sample can avoid the use of time consuming pre-enrichment steps to reach the limits required by some legislations that establish more restrictive detection limits (from 100 to 1000 CFU L^−1^). Moreover, the approach is able to clearly detect as low as 0.6 CFU mL^−1^ with a signal to background ratio of 9.7 (*n*=3) (Fig. 5, B).

This approach allows the determination of low concentrations in high-volume samples that is one of the bottlenecks in the detection of environmental bacteria, in around 2.5 h. As shown in Fig. 5, the LOD of the electrochemical immunosensor without filtration (only IMS) resulted in 100 CFU mL^−1^ improved to up to 0.1 CFU mL^−1^ when the preconcentration strategy was applied in 1 L of sample (10^3^ fold improvement). The values of the LODs and the total assay time for the electrochemical immunosensor combining filtration are summarized in Supp. data (Table S7). The readout time is also reduced to up to 3 min, based on the incubation of the enzymatic substrates (H_2_O_2_ and HQ) with a total of 2 min and the electrochemical measurement of less than 1 min. This represents a 10-fold reduction in time in comparison with an immunoassay with optical readout, where the substrates require a minimum incubation time between 15 and 30 min [22]. In previous work, we explored several strategies to achieve the LOD, such as incorporating a short preincubation step [17] or amplifying the genetic material after the IMS [16]. In the present study, we have achieved a remarkable 10^3^-fold improvement in the LOD, by combining filtration and IMS. Compared with other electrochemical immunosensors and genosensors previously described for *Legionella* or other Gram-negative bacteria (and summarized on Table 1), the results confirm the promising features of combining filtration, immunomagnetic separation and electrochemical immunosensing for the ultrasensitive detection of *Legionella* using commercial screen-printed electrodes.

**Table 1:** An overview of electrochemical biosensing approaches for the detection of *Legionella* and other Gram-negative bacteria

Method (genosensing/immunosensing) Bacteria	Electrochemical measurement	Assay time	Preconcentration/pre-enrichment/amplification	Specificity	LOD	Ref
Electrochemical genosensor for the detection of *Legionella* spp*.* (16S rRNA sequence) that integrates a loop-mediated isothermal amplification (LAMP)	Cyclic voltammetry	30 min	YES -Filtration of 250 mL water samples and elution of the DNA - loop-mediated isothermal amplification	YES 16 strains of *Legionella* tested 15 strains of non-*Legionella* species tested	10 fg of *Legionella* nucleic acids (NA), corresponding to 2 copies of the bacteria In water samples / 30 CFU 250 mL^−1^	[27]
Electrochemical genosensor for detection of the hybridization and target sequence (*mip* gene of *L. pneumophila*), using gold nanoparticles (Au NPs) supported by cysteamine (Cys A) electrodeposited on the surface of gold electrodes	Square wave voltammetry	27 min (electrode preparation NOT INCLUDED)	NO No tested with *Legionella*	Mismatched DNA	LOQ 1 z-molar of synthetic DNA coding for *mip* gene	[[28]
Signal-off double probe electrochemical genosensor for the detection of *Legionella* spp. and *L. pneumophila* by monitoring the DNA hybridization and enzymatic cleavage with the enzyme *EcoRI* in gold electrodes	Differential pulse voltammetry	50 min (PCR NOT INCLUDED)	YES Asymmetric PCR	Mismatched DNA DNA from *E. coli*, *K. pneumoniae, P. aeruginosa*, *S. pneumoniae*, and *S. aureus*	1.2 × 10^−9^ M of synthetic DNA	[29]
Electrochemical genosensor based on nanoporous alumina membrane for the detection of *Legionella* spp. (21-mer DNA sequence)	Differential pulse voltammetry	Approx 45 min (PCR NOT INCLUDED)	YES Asymmetric PCR	mismatched DNA	3.1 × 10^−13^ M of synthetic DNA coding for 21-mer gene	[30]
Electrochemical genosensor based on amplified molecular beacon for the detection of *Legionella* spp. (21-mer DNA sequence) using thiolated hairpin DNA-ferrocene probes on gold electrodes	Differential pulse voltammetry	45 min approx (PCR NOT INCLUDED)	YES PCR	Mismatched DNA	2.3 × 10^−14^ M of synthetic DNA coding for 21-mer gene	[31]
Microfluidic electrochemical genosensor for the detection of target sequence (*L. pneumophila*) based on two-step sandwich with strep-coated paramagnetic microparticles, a biotinylated capture probe and strep-AP (streptavidin-alkaline phosphatase conjugate)	Amperometry	16 min (PCR NOT INCLUDED)	NO	Mismatched DNA	0.33 nM of synthetic DNA	[32]
Electrochemical genosensor for the detection of the target sequence (*mip* gene sequence) of *L. pneumophila* based on a multiwall carbon nanotube (MWCNT) electrode	Differential pulse voltammetry	40 min (PCR NOT INCLUDED)	NO	Mismatched DNA	10 pM of synthetic DNA coding for *mip* gene	[33]
Electrochemical immunosensor for the detection of *L. pneumophila* developing an immunoassay in nitrocellulose filters	Amperometry	2–3 h	YES Filtration of 200 mL	(The antibody used in this paper was previously tested in another work)	4 CFU mL^−1^	[34]
Electrochemical immunosensor based on a microfluidic assay in water samples for the detection of *L. pneumophila*	Square wave voltammetry	100–180 min (depending on the detection mode, dynamic or static)	NO	NO	10 CFU mL^−1^	[20]
Electrochemical magneto immunosensor for the detection of *L. pneumophila* based on disposable core-shell Fe_3_O_4_@poly(dopamine) magnetic nanoparticles and a sandwich enzyme-linked immunosorbent assay	Amperometry	3 h	YES Filtration of 1 L of sample/ vortexing / centrifugation / Immunomagnetic separation	YES *E. coli*, *E. faecalis*, *P. aeruginosa*, *A. baumannii*, *Aeromonas* and *Salmonella*	10 CFU mL^−1^	[21]
Electrochemical immunosensor for the detection of *L. pneumophila* based on indium-tin oxide (ITO) electrodes	Electrochemical impedance spectroscopy	Not detailed	NO	The antibody used in this paper was previously tested in another work	50 CFU mL^−1^	[35]
Electrochemical immunosensor for the detection of PAL (peptidoglycan-associated lipoprotein, immune-dominant component of *Legionella* antigens) based on a ZnO nanorod (NR) matrix in gold electrodes and a sandwich enzyme-linked immunosorbent assay	Cyclic voltammetry	4 h	NO (The detection is done directly with PAL (peptidoglycan-associated lipoprotein) as an antigen of *L. pneumophila*)	PAL antigen (peptidoglycan-associated lipoprotein) as an antigen of *L. pneumophila*	∼1 pg mL^−1^ of PAL antigen	[36]
Electrochemical immunosensor for the detection of *L. pneumophila* based on a gold immunochip using fluorophore-conjugated antibodies	Impedance	1 h	NO	NO	2.0 × 10^2^ bacteria mL^−1^	[37]
Electrochemical genosensor for the detection of pathogenic bacteria including S*. enterica*, *L. monocytogenes*, and *E. coli* using silica particles and a triple-tagging PCR	Amperometry	3 h (PCR INCLUDED)	YES triple-tagging PCR	NO	0.04, 0.13 and 0.05 ng mL^−1^ for *S. enterica, L. monocytogenes* and *E. coli* target DNA respectively	[38]
Electrochemical genosensor for the detection of *E. coli* and *S. enterica* using strepAv-magnetic particles and quadruple-tagging PCR	Amperometry	2 h (PCR INCLUDED)	YES quadruple-tagging PCR	NO	0.59 and 0.75 pg μL^−1^ for *E.coli* and *S. enterica* respectively	[39]
Electrochemical genosensor for the detection of *Salmonella* combining phagomagnetic separation, double-tagging PCR and immunomagnetic separation	Amperometry	4 h (PCR INCLUDED)	YES Immunomagnetic separation / double-tagging PCR	YES *E. coli K12*	3 CFU mL^−1^	[18]
Electrochemical genosensor for the detection of *M. bovis* in milk using double-tagging PCR and streptavidin-magnetic beads	Amperometry	1 h 35 min (PCR NOT INCLUDED)	YES double-tagging PCR	NO	10 fmol of target DNA	[40]
Electrochemical immunosensor for the detection of *Salmonella* in milk samples using magnetic particles	Amperometry	50 min	YES Immunomagnetic separation / Optional: pre-enrichment of 8 h	YES *E. coli*	1,4 CFU mL^−1^ 0,108 CFU mL^−1^ (with pre-enrichment)	[17]
Electrochemical magneto-immunosensor for the detection of *L. pneumophila* retained in filters	Amperometry	<2.5 h	YES Filtration of 100 and 1000 mL / immunomagnetic separation of the bacteria retained in filters	YES *P. aeruginosa*, *K. pneumoniae*, *M. fortuitum*, *Enterobacter* spp., *E. coli*, *S. cholerasuis*	2 CFU mL^−1^ (100 mL) 0.1 CFU mL^−1^ (1000 mL)	This work

The electrochemical immunosensor described here allowed the determination of very low concentrations of *Legionella* in less than 2.5 h preventing time-consuming pre-enrichment steps filter treatments or DNA amplification. Further studies will focus on reducing the incubation time and streamlining it into a single step to simplify the analytical procedure.

## Conclusions

A magneto-actuated electrochemical immunosensor is presented in this work capable of detecting and quantifying *Legionella* in contaminated water in less than 2.5 h with an impressive LOD of 0.1 CFU mL^−1^. The most remarkable aspect of this approach is the elimination of classical pre-enrichment steps, DNA extraction, and amplification techniques, resulting in the analytical simplification and a significant reduction in the total analysis time. This innovative method combines a preconcentration strategy based on filtration to perform the IMS directly on polycarbonate filters. This allows for the handling of high-volume samples, typically ranging from 100 to 1000 mL. The electrochemical readout enables the use of a portable and cost-effective device that is compatible with in-field test. This represents the lowest reported LOD for the electrochemical biosensing of whole *Legionella* bacteria without the need for pre-enrichment and DNA amplification steps. The LOD achieved by this method underscores its utility, especially considering that some of the most stringent regulations require a LOD of at least 100 CFU L^−1^. Another noteworthy fact relies on the analytical simplification. In the gold standard microbiological method, after sample filtration, the retained bacteria were resuspended or extracted from the filter using special buffers or vortexing. In this study, the preconcentration strategy is integrated to the electrochemical biosensing. By filtering high volumes of samples and subsequently employing magnetic actuation to pull out the bacteria directly from the filter, the need for resuspension or additional steps for downstream analysis was eliminated. Another remarkable aspect of this approach is the potential of the magnetic particles of successfully removing the bacteria directly from various filtering materials. This promising characteristic broadens the applicability beyond water systems to include the detection of bacteria retained in air conditioning unit air filters, accomplished through direct immunomagnetic separation within the filters.

## Supplementary information

ESM 1 (PDF 6147 kb)

## Data Availability

The datasets generated during the current study are available in the CORA RDR repository, https://dataverse.csuc.cat/.
