# Supplementary material for: Improved biosensing of Legionella by integrating filtration and immunomagnetic separation of the bacteria retained in filters
Source: Mikrochim Acta. 2024 Jan 9;191(2):82. doi: 10.1007/s00604-023-06122-1 (PMC10774190; doi:10.1007/s00604-023-06122-1)
Supplement: Supplementary file 1 — (PDF 6147 kb) [file 604_2023_6122_MOESM1_ESM.pdf]

Supplementary material

IMPROVED BIOSENSING OF *LEGIONELLA* BY INTEGRATING FILTRATION AND IMMUNOMAGNETIC SEPARATION OF THE BACTERIA RETAINED IN FILTERS

Melania Mesas Gómez[a,b], Bárbara Molina-Moya[d,e], Bárbara de Araujo Souza[c], Maria Valnice Boldrin Zanoni[c], Esther Julián[f], José Domínguez[d,e,f], Maria Isabel Pividori[a,b*]

[a]Grup de Sensors i Biosensors, Departament de Química, Universitat Autònoma de Barcelona

[b]Biosensing and Bioanalysis Group, Institute of Biotechnology and Biomedicine, Universitat Autònoma de Barcelona, Bellaterra, 08193, Spain

[c]Department of Analytical Chemistry, Institute of Chemistry, UNESP, Universidad Estadual Paulista, Araraquara, SP, Brazil

[d]Institut d'Investigació Germans Trias i Pujol (IGTP), Badalona, 08916, Spain

[e]CIBER Enfermedades Respiratorias, Instituto de Salud Carlos III; Departament de Genètica i Microbiologia, Universitat Autònoma de Barcelona

[f]Departament de Genètica i Microbiologia. Universitat Autònoma de Barcelona

* Corresponding author. Grup de Sensors & Biosensors, Unitat de Química Analítica, Universitat Autònoma de Barcelona, Edificio Cn. Campus UAB, 08193, Bellaterra, Barcelona, Spain.

E-mail address: isabel.pividori@uab.cat (M.I. Pividori).

* Corresponding author. Grup de Sensors & Biosensors, Unitat de Química Analítica, Universitat Autònoma de Barcelona, Edificio Cn. Campus UAB, 08193, Bellaterra, Barcelona, Spain.

E-mail address: isabel.pividori@uab.cat (M.I. Pividori).

S1. Chemicals and biochemicals

Buffer and solution composition

The composition of the buffers used in this work are described below:

For covalent immobilization of antibodies on the magnetic particles

- Borate buffer: 100 mmol L^{-1} H_3BO_3, pH 9.0
- Ammonium sulphate buffer: 3 mol L^{-1} $(NH_4)_2SO_4$ dissolved in 100 mmol L^{-1} H_3BO_3 pH 9.0
- Blocking buffer: 500 mmol L^{-1} glycine dissolved in PBS pH 8.0
- Washing buffer: 10 mmol L-^{1} Na_2HPO_4, 0.137 mol L^{-1} NaCl, 0.1% (w/v) BSA
- Storage buffer: 10 mmol L-^{1} Na_2HPO_4, 0.137 mol L^{-1} NaCl, pH 7.4 plus 0.05% (v/v) Tween 20®, 0.05% (w/v) sodium azide

For sample incubation and washing steps

- PBS: 10 mmol L-^{1} Na_2HPO_4, 0.137 mol L^{-1} NaCl, pH=7.4
- Washing buffer: 10 mmol L-^{1} Na_2HPO_4, 0.137 mol L^{-1} NaCl, 0.5% (w/v) Casein

For electrochemical readout

- ePBS: 100 mmol L-^{1} Na_2HPO_4, 100 mmol L-^{1} KCl, pH 7.0.
- Electrochemical readout substrate solution: 57 mmol L^{-1} hydrogen peroxide, 23 mmol L^{-1} hydroquinone in distilled water

Legionella pneumophila culture

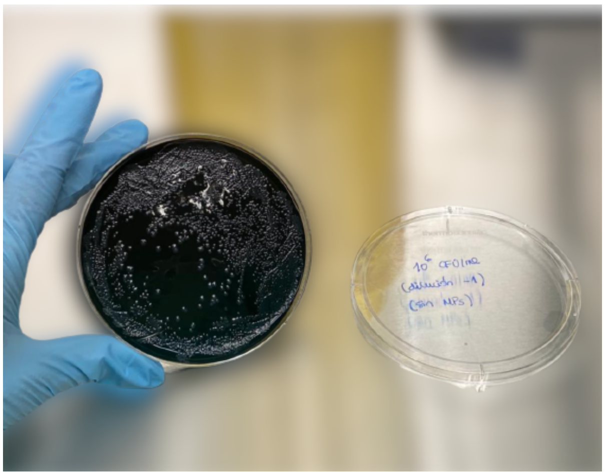

Figure S1. *Legionella pneumophila* strain (serogroup 1. Philadelphia 1. ATCC strain nº 33152) was grown in selective solid culture plates (*Legionella* MWY Selective Agar, Product no. 10482513, Thermo Scientific). The concentration of *Legionella* samples was calculated for each experiment by solid culture for colony counts verification.

S2. Covalent immobilization of anti-Legionella antibody

Covalent immobilization of anti-Legionella antibodies was performed in tosyl activated magnetic particles. The outline of the procedure is schematically represented in Figure S2, panel A. Briefly, a volume of 40 μL of MPs was washed twice with 1 mL of borate buffer followed by the addition of 8 μg of antibody and 100 μL of ammonium sulphate buffer performing a total volume of 250 μL.

The MPs were incubated under continuous agitation for a total reaction time of 20 h at 37ºC. After incubation, MPs were separated with a magnet and resuspended in 250 μL of 500 mM glycine for 2.5 h at 37ºC under continuous agitation to block the unreacted tosyl groups. The prepared anti-Legionella-MPs were washed three times with 1 mL of washing buffer and were resuspended in 160 μL of storage buffer at 4ºC. The final concentration of the anti-Legionella MPs was 1 x 10^8 MP mL^{-1}. The modified MPs were washed three times with a washing buffer before being used in each experiment.

After the immobilization, the supernatant is collected for the determination of the total amount of antibody immobilized on the magnetic particles by ELISA, providing a coupling efficiency of 95 % (represented in Figure S2, panel B).

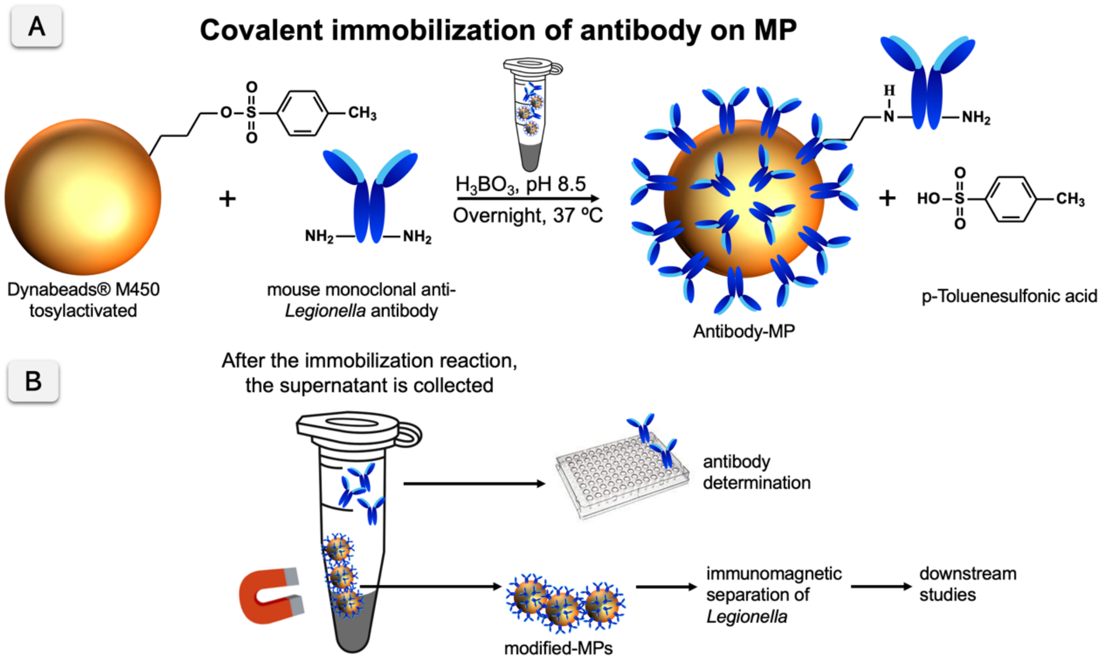

Figure S2. Schematic representation of the tailored covalent immobilization of antibodies on magnetic particles, followed by the determination of the coupling efficiency by determining the remaining antibodies on the supernatant by ELISA.

Afterwards, the total immobilization in percentages (coupling efficiency) is calculated based on the following formula, providing a value of 95 %.

$$\text{Immobilization (\%)} = \frac{[\text{ab initial}] - [\text{ab supernatant}]}{[\text{ab initial}]}$$

Magnetic separation by using the magnetic separator is performed after each incubation/washing step to separate the magnetic particles from the supernatant.

S3. Electrochemical magneto immunosensing. Optimization of the reagent concentrations

The reagent concentrations for the magneto-actuated immunosensor were optimized in order to obtain the maximum signal-to-noise ratio.

The concentration of the modified magnetic particles with anti-Legionella antibodies and the anti-Legionella-HRP antibody were optimized using an optical readout (magneto-actuated immunoassay).

On the one hand, in a 2.0 mL Eppendorf tube, a volume of 800 µL of PBS, 100 µL of positive or negative controls (1.1 x 10^6 and 0 CFU mL^{-1} of *L. pneumophila,* respectively) and 100 µL of anti-Legionella-MPs at different concentrations of 10^4, 10^5, 10^6, 10^7 MP mL^{-1} were tested. Then, after 1 h incubation, the supernatant was discarded using a magnet and 1 mL of washing buffer was added to perform one washing at the rotor. Then, the sample was resuspended in 200 µL and it was washed twice in a thermomixer with 200 µL of washing buffer. Afterwards, 200 µL of anti-Legionella-HRP at 1/2000 dilution was incubated for 30 min at the thermomixer for 30 min at 900 rpm and RT. The sample was washed twice again, resuspended in 200 µL and transferred to a magneto-ELISA microplate (polypropylene microplate). One washing was done in the microplate with 200 µL, and 100 µL of the optical substrates TMB/H_2O_2 were added for 30 minutes in continuous agitation. Finally, the reaction was stop with 100 µL of H_2SO_4 2M and the absorbance was read at 450 nm to compare the signals (Figure S3, Panel A). For the optimization of the anti-Legionella-HRP concentration, the same procedure described above was followed, using 10^7 MP mL^{-1} as modified MPs concentration. Then, the dilutions including 1/250, 1/500, 1/1000 and 1/2000 were tested for the anti-Legionella-HRP antibody (Figure S3, panel B). The optimized concentrations for further steps were 10^7 MP mL^{-1} and a dilution of 1/500 for the anti-Legionella-MPs and anti-Legionella-HRP antibodies, respectively.

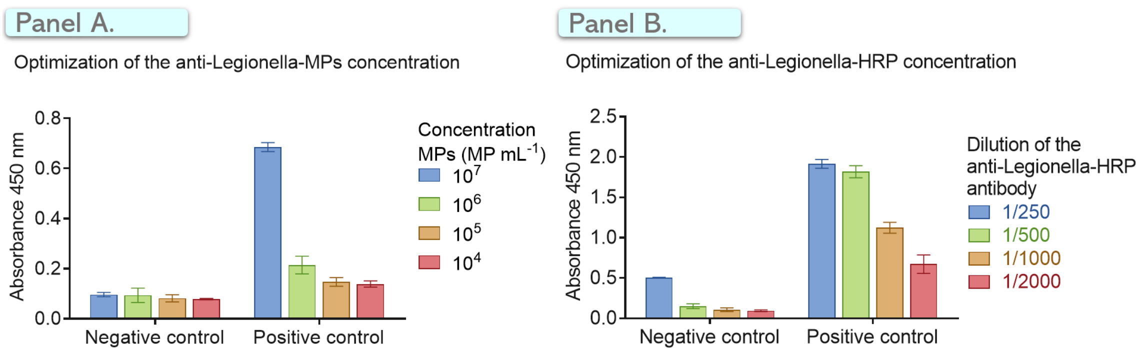

Figure S3. **Panel A)** Bar plot for the optimization of the anti-Legionella-MPs (n=3). L. pneumophila 1.1 x 10^6 CFU mL^{-1}**. Panel B)** Bar plot for the optimization of the anti-Legionella-HRP antibody concentration (n=3). L. pneumophila 4.9 x 10^6 CFU mL^{-1}. In all instances, negative controls were also assessed.

S4. Electrochemical magneto immunosensing for the quantification of *Legionella pneumophila*

The conditions for the electrochemical immunosensing were previously optimized as described in S3 (Supp. data). From the results, a concentration of 10^7 anti-Legionella-MPs mL^{-1} and anti-Legionella-HRP antibody 1/500 was used in all further experiments.

Figure S4 shows the calibration plot from 0 to 3.6 x 10^5 CFU mL^{-1} for the determination of the *L. pneumophila* with the electrochemical immunosensor without the integration of the novel preconcentration method. The data was fitted with a non-linear regression (Sigmoidal 4PL, GraphPad Prism Software v 10.0.1, R^2= 0.9837) and the Limit of Detection (LOD) was calculated, resulting in a value of 100 CFU mL^{-1}.

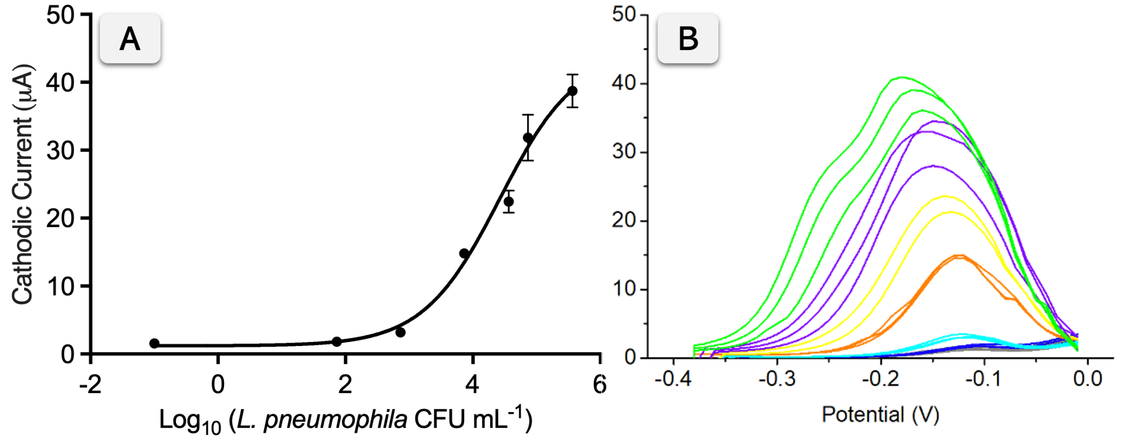

Figure S4. Panel A. Calibration plot for the magneto-actuated electrochemical immunosensor in water samples. The black solid line shows the calibration plot ranging from 0 to 3.6 x 10^5 CFU mL^{-1} without the integration of the novel preconcentration method (R^2= 0.9837). The concentration of the viable bacteria was

estimated by culturing in solid media and further CFU counting. The error bars show the standard deviation (n=3). Panel B. Raw data obtained from the SWV measurements for the magneto-actuated electrochemical immunosensor. The potential range was 0 to -0.4 V, with a potential step and amplitude of 10 mV, and frequency of 1 Hz. The maximum current value was used for the calibration plot.

S5. Electrochemical readout

The electrochemical readout was based on square wave voltammetry using carbon screen-printed electrodes (ref. DRP-C110, DropSens, Spain). The electrochemical cell consists of a working and auxiliary electrode made of carbon, while the reference electrode is made of silver, and a permanent neodymium magnet was placed above the electrode (Figure S4, panel A). These electrodes are of small dimensions (L 33 x W 10 x H 0.5 mm) and allow working both with sample microvolumes or even in solution. The electrodes were connected to the boxed connector for SPE (Ref. DSC) which operates as an interface between the electrodes and the portable bipotentiostat (DRP-STAT200, DropSens, Spain). The square wave voltammetry (SWV) measurements were performed in a laptop computer in which the portable bipotentiostat was connected by a universal USB port. The anti-Legionella antibodies labeled with the HRP enzyme, were used as electrochemical reporters in the presence of hydrogen peroxide (H_2O_2) as a substrate of HRP and hydroquinone (HQ) as a mediator. HRP catalyze the transfer of two electrons from HQ to H_2O_2 to generate water and benzoquinone (BQ), the oxidized form of HQ. First, the HRP is oxidized catalyzing the reduction of H_2O_2 to H_2O, then the HRP is reduced again by oxidizing HQ to BQ. Thus, the final readout at the surface of the electrode is based on the reduction of the BQ to HQ (Figure S4, panel B). Thus, since the enzyme works at saturated substrate conditions, HRP works at maximum speed and turn-over range, providing a directly proportional signal to the sample (hence the higher the number of bacteria with anti-Legionella-HRP, the higher the signal).

The SWV measurements were performed with a total of 62 µL of sample. The sample performed by the modified MPs capturing the bacteria and the HRP enzyme labelled antibody was resuspended in 40 µL of ePBS, and 18 µL of the electrochemical readout substrate solution containing hydrogen peroxide and hydroquinone was added. After the homogenization, the enzymatic reaction was done in 2 min and the solution was transferred to the surface of the screen-printed electrode. The potential range used was from 0 to -0.4 V, with a potential step and amplitude of 10 mV, and frequency of 1 Hz. The data were recorded and processed using DropView200 software.

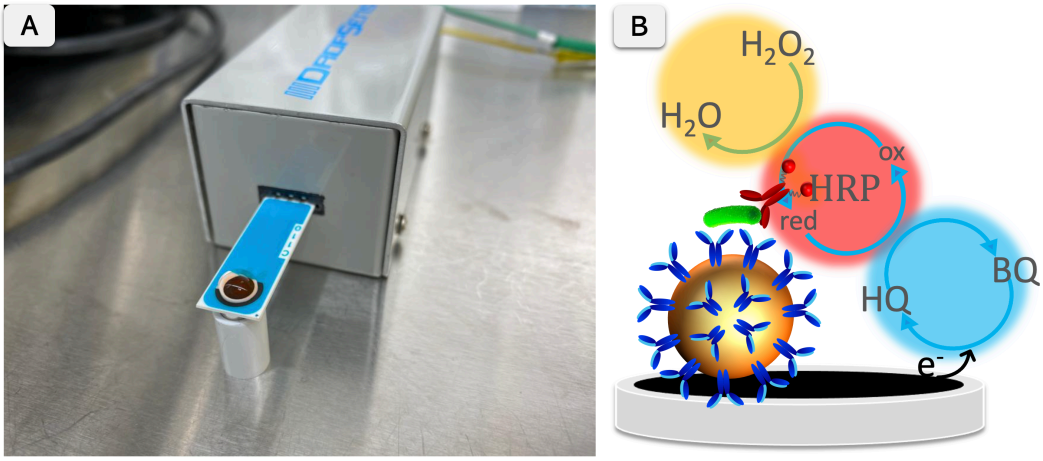

Figure S5. Schematic representation of the electrochemical set-up and the HRP enzymatic reaction on the electrode surface, to achieve the electrochemical readout.

S6. Novel preconcentration method. Study of the filtering material

Briefly, the procedure combines three steps as described in Figure 1 and S5: i) Filtration of large volumes of sample (typically 100 or 1000 mL), followed by ii) immunomagnetic separation of the bacteria retained in the filter and magnetic actuation, iii) electrochemical immunosensing, as described above.

In detail, following the filtration from 100 to 1000 mL sample under vacuum (Figure S5, panel A), the 25 mm diameter filter was placed on a 2.0 mL tube (panel B). 100 µL of anti-Legionella-MPs at 10^7 MP mL^{-1} and 900 µL of PBS were then added to the filter (panel C) and incubated under gentle rotation at RT for 1 h (panel D). Then, the MPs with the captured *Legionella* were recovered under magnetic actuation by means of a permanent magnet (panels F to I) and washed for 3 minutes at 900 rpm and RT. After that, 200 µL of the anti-Legionella-HRP antibody was added to the modified MPs and incubated for 30 min at 900 rpm and room temperature. After washing, the electrochemical readout was performed as described above.

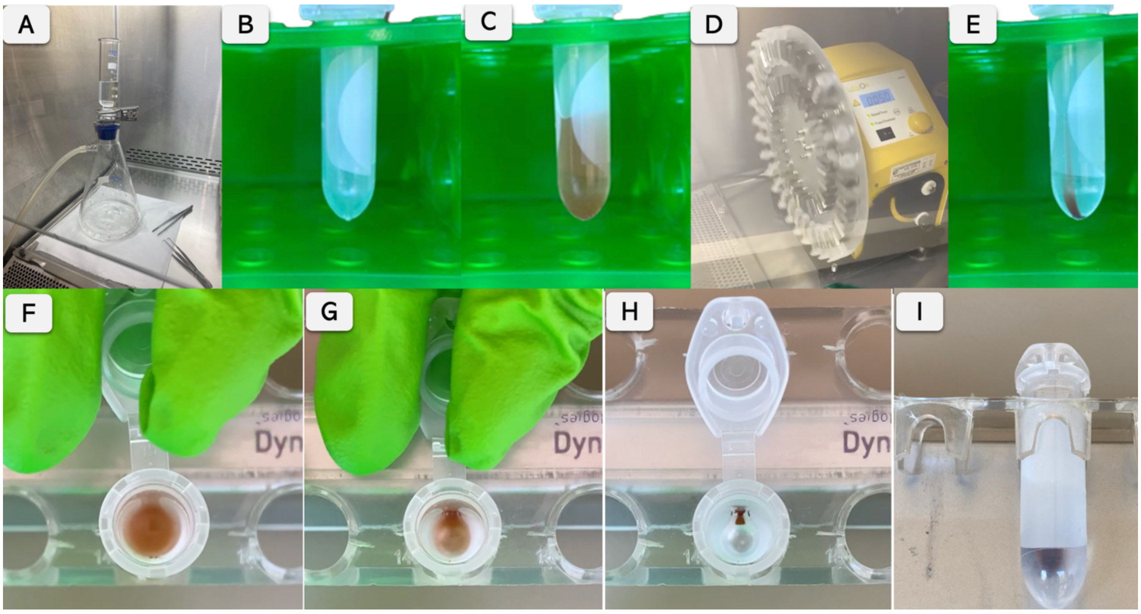

Figure S6. Schematic representation of A) Filtration, B to D) immunomagnetic separation of the bacteria retained in the filters, showing how the filter is positioned on the Eppendorf tube for immunomagnetic separation (B and C) under rotation (D) and (F to I) magnetic actuation. The photos (F to I) were captured in 1-second frame sequences.

The workflow time was calculated for each filtering material and the values are described in table S6. The LOD of the electrochemical immunosensor without the integration of the novel preconcentration method, and the electrochemical immunosensor with the integration of filtration of 100 and 1000 mL and IMS are summarized in table S7.

Table S6. Workflow time by using different filtering materials: nylon (NY), mixed cellulose ester (MCE), cellulose nitrate (NC), cellulose acetate (CA) and polycarbonate (PC), by the filtration of 50 mL sample and time required for 100 mL samples.

FILTERING MATERIAL	Nylon	Mixed cellulose ester	Cellulose nitrate	Cellulose acetate	Polycarbonate
Workflow time (50 mL sample)	74 s	23 s	38 s	45 s	22 s
Approximate filtration time (100 mL sample)	>2 min	< 1 min	> 1 min	> 1 min	< 1 min

S7. Summary of the analytical performance

The LOD of the electrochemical immunosensor without the integration of the novel preconcentration method, and the electrochemical immunosensor with the integration of filtration of 100 and 1000 mL and IMS are summarized in table S7.

Table S7. Summary of the results obtained for the electrochemical immunosensor including sample volume processed, LOD and number of replicates of a zero calibrator or blank sample used to calculate the LOD, and the total assay time.

	**Without preconcentration strategy**	**With preconcentration strategy**	
**Sample volume (mL)**	0.1	100	1000
**LOD (CFU mL^{-1}) / number of blank sample (replicates)**	1 x 10^2 / n=8	2 / n=11	0.1 / n=3
**Total assay time**	~2h 15 min	~2h 17 min	~2h 26 min
